# Can the supplementation of vitamin D, sun exposure, and isolation during the COVID-19 pandemic affect the seasonal concentration of 25(OH)D and selected blood parameters among young soccer players in a one-year training season?

**DOI:** 10.1080/15502783.2023.2206802

**Published:** 2023-05-03

**Authors:** Joanna Jastrzębska, Maria Skalska, Łukasz Radzimiński, Guillermo F. López Sánchez, Lee Hill, Katja Weiss, Beat Knechtle

**Affiliations:** a Diabetology and Endocrinology, Department of Pediatrics, Gdansk Medical University, Department of Pediatrics, Gdansk, Poland; b Department of Health and Natural Sciences, Gdansk University of Physical Education and Sport, Department of Health and Natural Sciences, Gdansk, Poland; c Division of Preventive Medicine and Public Health, Department of Public Health Sciences, School of Medicine, University of Murcia, Division of Preventive Medicine and Public Health, Department of Public Health Sciences, School of Medicine, Murcia, Spain; d Department of Pediatrics, Division of Gastroenterology and Nutrition, McMaster University, Department of Pediatrics, Division of Gastroenterology and Nutrition, Hamilton, ON, Canada; e Institute of Primary Care, University of Zurich, Institute of Primary Care, Zurich, Switzerland; f Medbase St. Gallen Am Vadianplatz, St. Gallen, Switzerland

**Keywords:** Supplementation, football, home-based training, COVID-19

## Abstract

**Objective:**

This study examined the effect of vitamin D supplementation, sunlight radiationradiation, and home isolation during the COVID-19 pandemic on the seasonal changes in 25(OH)D concentration and selected biomarkers in young soccer players along a one-year training cycle.

**Method:**

Forty elite young soccer players (age: 17.2 ± 1.16 years, body mass: 70.2 ± 5.84, and body height: 179.1 ± 4.26 cm) participated in the research. Only 24 players completed the measurements during all four time- points (T1-: September 2019, T2-: December 2019, T3-: May 2020, and T4-: August 2020) and were divided into two subgroups: supplemented group (GS) and placebo group (GP). Players from GS received 5,000 IU of vitamin D for 8 weeks (January-MarchJanuary–March 2020). Several biomarkers such as 25(OH)D, white blood cells (WBC), red blood cells (RBC), hemoglobin (HGB), muscle damage markersmarkers, and lipid profile were measured.

**Results:**

AnalysisThe analysis of the total group demonstrated significant seasonal changes in 25(OH)D, HGB, asparagine aminotransferaseaminotransferase, and creatine kinase along the one1-year training cycle. The level of 25(OH)D concentrationinconcentration in T4 was significantly (*p* < 0.001, pη [ = 0.82) higher in both subgroups in comparison to T2 and T3. Moreover, the significant (*p* = 0.023) but poor (*r* = −0.23) correlation between 25(OH)D and WBC was calculated.

**Conclusion:**

Current research confirmed the significant seasonal changes in 25(OH)D concentration during four seasons. 8-weekEight-week vitamin D supplementation had no extended effect on the level of 25(OH)D concentration.

## 1. Introduction

The seasonal changes in 25(OH)D (vitamin D) concentration in blood plasma mainly concernsconcern the inhabitants of countries where the sunlight radiation varies according to the season (e.g. Northern Europe, Canada,Canada, and northern states of the USA) [2]. The largest deficits of 25(OH)D have been observed in people from these areas between October and April when the ultraviolet (UV) index does not exceed 2–3 and the number of sunny days is relatively low. Thus, maintaining the optimal level of this metabolite in blood plasma requires a diet focused on food products rich in vitamin D, appropriate supplementation [1], and exposure to UV rays [3–5]. Interestingly, in the countries of North Africa or the Mediterranean basin, a low level of 25(OH)D concentration was also reported. However, these shortages could be explained by the protection against overexposure to sunlight or the use of sunscreen [6,7].

Several authors [8–10] reported 25(OH) shortages and deficits during autumnduring the autumn and winter in soccer players from southern Poland. Similar seasonal changes were demonstrated in young Russian soccer players [11], English students [12], and Swiss athletes from different sports [13]. In contrast, Brustad et al. [14] reported that a diet rich in vitamin D might improve its level regardless of the season. The authors stated that high dietary intakes of vitamin D (including fish) mask the effect of seasonal changes in UV exposure during winter.

Vitamin D supplementation in young athletes is one of the most important factors protecting against its deficits. Skalska et al. [15] claimed that a training load analysis during the experiment is an important aspect when investigating the effect of supplementation on the biomarkers. It is essential that both experimental and control groups follow the same training program.

Numerous studies have analyzed the correlations between seasonal variations in vitamin D [16,17], the effect of its supplementation on physical performance [18–20], and bone resorption markers [8,21,22]. Nevertheless, the number of researches investigating these topics with reference to blood parameters is still scarce [23,24].

The type of sport is another important factor determining the amplitude of changes in 25(OH)D concentration. The essential issue is whether the training sessions are conducted indoors or outdoors. The meta-analysis by Farrokhyar et al. [25] showed that the vitamin D deficiency at over 50% of athletes correlated with latitude, winter seasonseason, and indoor training sessions. This observation was confirmed by Maruyama-Nagao et al. [26], who found that more significant changes in this metabolite were reported in women training inside the sports hall than in women who performed their training in the open air.

When the COVID-19 pandemic was announced at the beginning of March, outdoor physical activity was prohibited due to the pandemic restrictions introduced in most European countries. As a result, numerous athletes were forced to perform home-based training [27]. Grazioli et al. [28] reported that home isolation significantly and negatively affected soccer players’ physical fitness levels. Furthermore, Hadizadeh et al. [29] suggested that vitamin D supplementation could decrease the deficiency caused by the lower insolation. According to Jimeno-Almazán et al. [30], the role of an optimal level of 25(OH)D could be essential to avoid post-COVID dysfunctions.

Due to the important role of vitamin D in physiological processes during physical effort in young athletes with deficiency of this biomarker during some seasons, the project considering its changes collectively with chosen blood parameters in young soccer players was introduced.

This research aimed to investigate the influence of vitamin D supplementation, sunlight radiationradiation, and home isolation during the COVID-19 pandemic on seasonal changes in 25(OH)D and selected biomarkers in young soccer players along a one1-year training cycle. It was hypothesized that due to the lower sunlight radiation, the highest drop in 25(OH) concentration will occur in the autumn and winter season. Therefore, vitamin D supplementation might effectively prevent its deficiency and negative changes in related blood parameters. However, the home-isolation factor was not part of the design because the COVID-19 pandemic occurred in March 2020 during the project.

## 2. Material and methods

This study is part of a larger scientific project. Therefore, the study design and some of the procedures were previously published in the papers of Jastrzębska et al. [31,32].

### 2.1. Participants

Forty elite young soccer players took part in the study. Unfortunately, due to the COVID-19 pandemic and other random occurrences, only 24 players completed the project (age: 17.2 ± 1.16 years, body mass: 70.2 ± 5.84, body height: 179.1 ± 4.26 cm, and BMI: 21.9 ± 1.7 kg/m^2^). All participants were members of the same team, competing in the Polish Central Junior League and students at the same private high school (sport class with the soccer profile). A minimum required attendance was set at the level of 85%. A majority of the players (74%) lived in the school dormitory and had a homogenous standard diet consisting of a large number of vegetables, fruits, and dairy products, overseen by the club’s dietitian. One month before the project began, no vitamin supplements (especially vitamin D) were provided. In addition, during the winter and autumn periods, the athletes wore full-body sportswear due to the low outside air temperatures. Therefore, the lack of UV exposure was one of the factors that could reduce the natural vitamin D synthesis.

**Figure 1.**
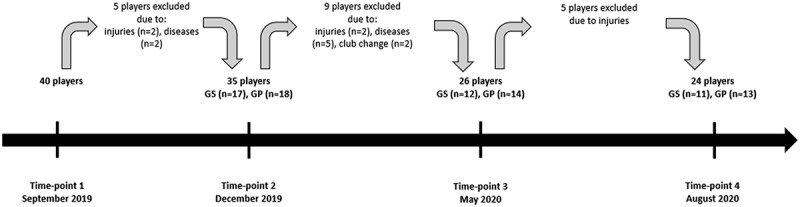
The study timeline and flowchart of the participants.

Injuries and illnesses prevented five players from continuing the research. The rest of the group (*n* = 35) was divided into a supplemented group (GS, *n* = 18) and a placebo group (GP, *n* = 17). The players were randomly assigned to the groups, and there were no significant differences in 25(OH)D concentration between GS and GP at baseline. This project stage was completed by 11 players from GS and 13 players from GP (Figure 1).

Ethical approval was received from the Local Bioethics Medical Committee in Gdansk (Poland) (KB consent number − 26/19). The study was conducted in accordance with the Helsinki Declaration of 2013. Following familiarization and explanation of the study objectives, procedures, and methodology, all participants consented to participate. All the participants or their legal guardians were requested to sign the informed consent form.

### 2.2. Inclusion and exclusion criteria

All the players were members of the same club and performed identical training loads. Only participants who completed at least 85% of training sessions and had no SARS-CoV-2 infection were included in the study. Moreover, players who used a diet other than the preferred diet of the club, including supplementation of vitamin D, and minerals (especially Calcium and Phosphorus)calcium and phosphorus), or made use of a solarium, used anti-solar filters, and trained outside without the allowance of the club trainers were excluded from the research. During a one1-year training season, a total of 16 players did not meet the inclusion criteria (6six players were injured, another 4four missed more than 15% of training sessions, and 6six players were excluded due to illness). Finally, 24 players were included in the analysis of the results.

### 2.3. Study design

The study employed a cross-sectional study design. The research was conducted on 40 young soccer players between the middle of September 2019 and the end of August 2020. It was assumed that the amount of sunlight where participants were staying would not influence the synthesis of vitamin D in the period from early autumn to the end of the winter. Moreover, the radiation of sun rays could increase vitamin D synthesis from the beginning of spring. During the analyzed period, all the players performed the same training program involving technical and tactical drills, official and friendly matches, or exercises developing aerobic capacity, speed, strength, and power. Within a one1-year training cycle, the volume and the intensity of the training varied depending on the part of the season. The competition season training process was planned according to the next game and included phases of intentionally increasing or decreasing the training load. A typical weekly training load in the competition period contained six training sessions for all players, three for individual training (defenders, middle players, and strikers)strikers), and one league game (12–15 h a week). During the preseason, a large number of general training drills were used along with the soccer-specific exercise. Therefore, the preparation period contained a higher training volume, and several drills were performed with high intensity (Table 1).

**Table 1.** Training load (min) taking into account type (overall and special drills) and intensity (aerobic, or anaerobic) of exercises of typical weekly training during competition and preparation period of one1-year training season of young soccer players.

	Aerobic performance (min)	Aerobic-–anaerobic performance (min)	Anaerobic lactate performance (min)	Anaerobic nonlactate performance (min)	Total (min)
**Competition period**					
Overall drills	225	30	5	20	280
Special drills	160	220	30	30	440
Total (min)	385	250	35	50	**720**
**Preparation period**					
Overall drills	250	80	25	35	390
Special drills	190	185	18	27	420
Total (min)	440	265	43	62	**810**

### 2.4. Time-point (T) measurements of 25(OH)D, blood parameters, and supplementation of vitamin D

The study design involved the measurements of 25(OH)D and blood parametersparameter concentration in blood plasma. Additionally, during the project, the number of sunny days (UV radiation) and the players’ diet were recorded. All the measurements were performed four times: MThe middle of September 2019 (T1), middle of December 2019 (T2), beginning of May 2020 (T3, the date was changed from the middle of March because of the COVD-19COVID-19 lockdown), and end of August 2020 (T4). Due to significant drops in the players’ 25(OH)D concentration, vitamin D supplementation was applied after T2. Therefore, the supplementation lasted from the middle of January to the middle of March (all preseason periods).

### 2.5. COVID-19 lockdown and home-based training

The COVID-19 lockdown occurred in Poland and other countries all over Europe and the world. From the middle of March 2020 to the middle of May 2020, the tested players were not allowed to perform team training sessions. The home-based training (usually performed indoors) was applied instead. This situation did not allow us to carry out the measurements according to the plan (immediately after completing the vitamin D supplementation). Thus, the T3 tests were conducted as soon as possible in the middle of May 2020. The fourth set of measurements was performed as planned at the end of August 2020 (Figure 1).

### 2.6. Degree of UV radiation

**Figure 2.**
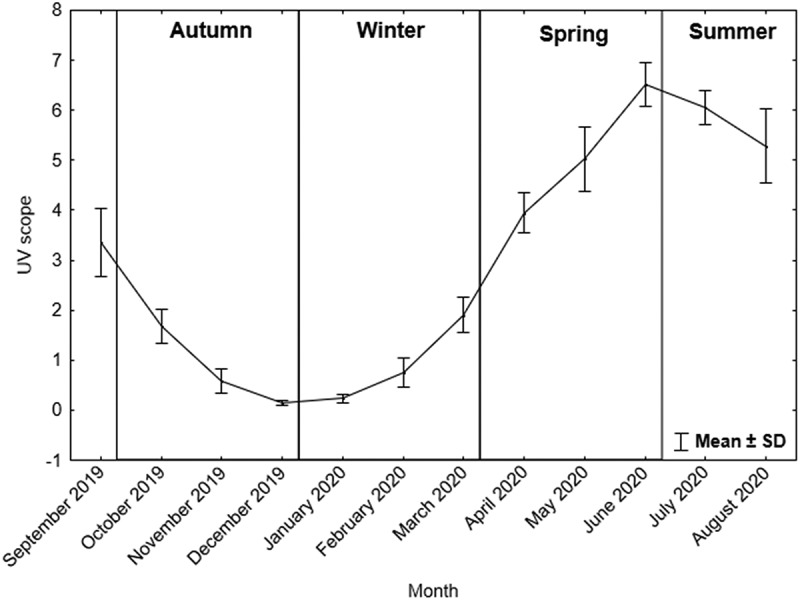
The scope of the UV index for the city of Gdynia (Poland) during the project [33].

The research was conducted in the northern part of Europe in Poland (Gdynia, 54.50° N 18.55° E, 33 m above sea level). The degree of UV radiation during the project duration, i.e., between September 2019 and August 2020, was recorded based on historical data from the weather online service for the city of Gdynia [33] (Figure 2).

### 2.7. Calculation of biochemical analyses and 25(OH)D

Biochemical analysis of blood was carried out in an accredited analytical laboratory Diagnostics-medical laboratoriesan accredited analytical laboratory Diagnostics-medical laborator,y, Gdynia, Poland). The Sysmex XE 2100 D and XT 4000 analyzers (Sysmex Europe GmBH, Norderstedt, Germany) were used to calculate blood serum selected parameters. WBC (white blood cells), RBC (red blood cells), HGB (hemoglobin), HCT (hematocrit), and PLT (thrombocytes) were calculated by fluorescent flow cytometry technology using reagents: Cellpack, Cellsheat, Stromatolyser FB, StrolamtolyserStromatolyser-4DL, Stromatolyser-4DS, and Sulfolyser. The intra-assay CV of the method and with respect to the range were respectivelywere, respectively, as forllows: WBC 3,.0% (at least 4,.0 × 10^3^/μl) and 4.0–10.0 10^3^[33]/µl; RBC 1,.5% (at least 4,.00 × 10^6^/μl) and 4.2–5.6 × 10^4^ [4]/µl; HGB 1,.0% and 12.1–16.6 g/dL; HCT 1,.5% and 35.0–49.0%; and PLT 4,.0% (at least 100 × 10^3^/μl) and 18–430 10^3^ [3]/μl.

TC (total cholesterol) was calculated by calorimetry method with cholesterol esterase reagent (Roche Cobas 6000). The intra-assay CV of the method was 1.6% with respect to the range 115.0–190.0 mg/dL. HDL-C (high-density cholesterol) was calculated by calorimetry method with HDLC4 reagent (Roche Cobas 6000). The intra-assay CV of the method was 2.2% with respect to the range ≥40 mg/dL. LDL-C (low-density cholesterol) was calculated by spectrophotometry method with LDLC3 reagent (Roche Cobas 6000). The intra-assay CV of the method was 2.5% with respect to the range 0–130 mg/dL. TG (triglycerides) was calculated by calorimetry method with Roche 11–2017 reagent (Roche Cobas 6000). The intra-assay CV of the method was 2.0% with respect to the range 0–150 mg/dL. ALT (alanine aminotransferase) was calculated by spectrophotometry method with Roche 09–-2018 reagent (Roche Cobas 6000). The intra-assay CV of the method was 3.3% with respect to the range 0–41 U/L. AST (asparagine aminotransferase) was calculated by spectrophotometry method with Roche 09–-2018 reagent (Roche Cobas 6000). The intra-assay CV of the method was 2.3% with respect to the range 0–40 U/L. CK (creatine kinase) was calculated by spectrophotometry with Roche 05–-2017 reagent (Roche Cobas 6000). The intra-assay CV of the method was 3.2% with respect to the range 39–308 U/L. The chemiluminescence (CMIA) (Liaison XL, Diasorin, Saluggia, Italy) using 25OH vitamin d total assay reagent was applied to calculate the serum concentration of 25(OH)D. The intra-assay CV of the method was 2.4–6.4%, with respect to the range; 0–20 ng/mL deficit, >20–30 ng/mL suboptimal concentration, >30–50 ng/mL optimal concentration, >50–100 ng/mL high, >100 ng/mL potentially toxic, and >200 ng/mL toxic [34].

### 2.8. Calculation of average vitamin D intake

Diet (V.6.0) software developed by the Institute of Nutrition (Poland, 2018) and a vitamin D calculator in food products were used to calculate the daily vitamin D intake for each player during the first and last weekweeks (from Monday to Sunday) of the competition and preparation period of thecprojectthe project.

### 2.9. Supplementation of vitamin D

Subjects from the supplemented group (GS) were given the vitamin D bottle (Vigantol Merck), whereas the non-supplemented group – placebo (GP) received identical bottles with sunflower oil. All participants were asked to take 10 droplets per day (vitamin D or sunflower oil). GS received 5,000 IU of vitamin D daily in the morning. Players and the person who administered the supplementation were blinded to the group’s assignment.

### 2.10. Statistical Analyses

The distribution of the data was verified using the Shapiro–Wilk test. The analysis of variance (ANOVA) for repeated measures was used to check potential differences between successive tests. The post-hoc Honestly Significant Difference (HSD) Tukey test for unequal samples was performed to identify which measurements’ significant differences appeared. A non-parametric Friedmans’ ANOVA with an appropriate post-hoc test was applied for the variables where distribution was inconsistent with normal distribution. Following the grouping into GS and GP, a two-factorial variance analysis (ANOVA) was performed for repeated measurements. This analysis was used to assess both inter-group and intra-group effects. Moreover, the post hoc observed power was calculated. A partial eta square (*p*η [1] was calculated to determine the effect’s magnitude. The *p*η [1] was classified as small (≥0.01), medium (≥0.06), and large (≥0.14) [35]. Additionally, the observed power (OP) was calculated. The possible relations between the blood parameters and the results of physical measurements were calculated using Pearson’s correlation. They were fixed into the following categories: very strong (*r* ≥ 0.80), moderately strong (*r* = 0.60–0.79), fair (*r* = 0.30–0.59), and poor (*r* ≤ 0.29). The significance of differences for all analyses was calculated at *p* < 0.05. The statistical analysis was performed using Statistica version 13.0 (TIBCO Software Inc., 2017 Palo Alto, CA, USA).

## 3. Results

The typical training volume in a competitive season was 720 minutes720 min a week and mainly involved aerobic intensity exercise (53.5% of totalthe total time) and mixed aerobic-–anaerobic intensity drills (34.7%). During the pre-season period (partially due to the sports camp occurrence), the weekly training volume increased to 810 minutes810 min. However, the percentage distribution of the training loads was similar to the in-season period (Table 1).

The scope of ultraviolet (UV) radiation during the analyzed period is presented in Figure 2. The highest values of UV radiation were noted from the beginning of May to the half of September.

The analysis of the selected blood parameters in the total groups of 24 players in four time-pointstime points from four different seasons demonstrated that the largest difference was in blood plasma’s 25(OH)D concentration. The highest level of this biomarker was noted at the final stage of the project (in the late summer). Moreover, this value was significantly (*p* < 0.05) higher than other time points. The statistical analysis of changes in other biomarkers indicated significant differences in HGB, HCT, ASTAST, and CK between different time points (Table 2).

**Table 2.** Changes in selected blood parameters in young soccer players (*n* = 24) during a one-year training cycle.

Time point	T1	T2	T3	T4
25(OH)D (ng/ml)	35.0 ± 6.26	24.5 ± 4.89*	26.4 ± 5.32	40.5 ± 6.86*^#^†
WBC (10^3^ [3]/µl)	5.84 ± 1.10	6.16 ± 1.09	5.76 ± 1.15	6.00 ± 1.11
RBC (10^4^ [4]/µl)	5.14 ± 0.29	5.29 ± 0.35	5.07 ± 0.30	5.25 ± 0.37
HGB (g/dL)	14.9±0.71	15.5 ± 0.73*	14.9 ± 0.61^#^	15.4 ± 0.73
HCT (%)	44.6 ± 1.92	45.8 ± 1.97	44.3 ± 1.84^#^	45.5 ± 2.24
PLT (10^3^ [3]/µl)	228.3 ± 39.84	229.5 ± 34.08	213.4 ± 34.28	221.0 ± 42.87
TC (mg/dL)	142.5 ± 21.83	145.9 ± 22.28	147.6 ± 22.03	146.4 ± 23/27
HDL-C (mg/dL)	55.6 ± 9.19	56.1 ± 8.47	56.8 ± 7.97	53.00 ± 8.41
LDL-C (mg/dL)	74.3 ± 18.26	69.5 ± 15.98	77.2 ± 20.67	76.8 ± 17.74
TG (mg/dL)	63.4 ± 17.37	95.6 ± 48.56	64.3 ± 18.56	84.6 ± 30.63
ALT (U/L)	18.5 ± 4.77	19.3 ± 8.83	21.5 ± 6.62	17.5 ± 4.99
AST (U/L)	27.5 ± 7.93	22.8 ± 5.96*	33.5 ± 12.04^#^	25.7 ± 6.57†
CK (U/L)	341.7 ± 172.9	180.8 ± 91.9*	564.3 ± 335.7^#^	271.6 ± 156.1†

*Significantly different from T1; ^#^Significantly different from T2; †Significantly different from T3; WBC-: white blood cells; RBC-: red blood cells; HGB-: hemoglobin; HCT-: hematocrit; PLT-: thrombocytes; TC-: total cholesterol; HDL-C-: high-density cholesterol; LDL-C-: low-density cholesterol; TG-: triglycerides; ALT-: alanine aminotransferase; AST-: asparagine aminotransferase; CK-: creatine kinase.

**Figure 3.**
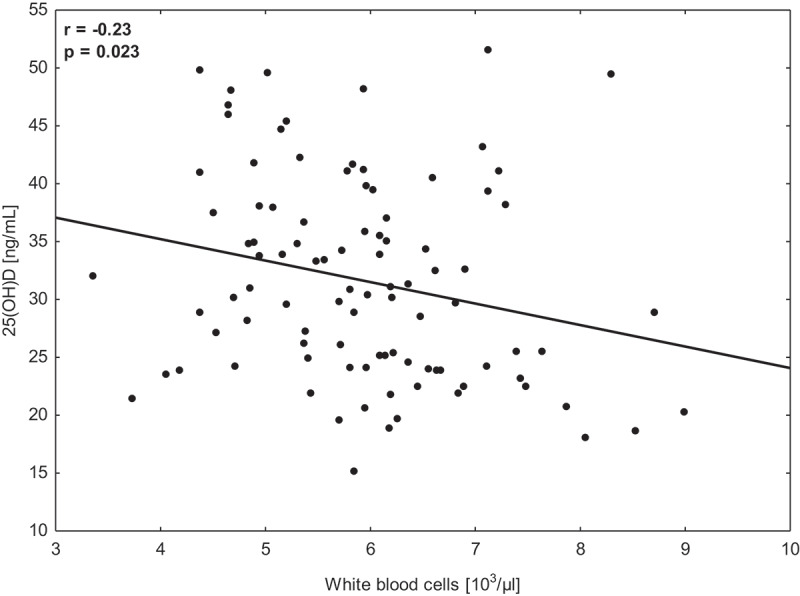
Correlations between 25(OH)D concentration and white blood cells in young soccer players (*n* = 24) during a one1-year training cycle.

The two-way ANOVA demonstrated the significant (*p* < 0.001, *p*η [1] = 0.82) time interaction for 25(OH)D concentration with no significant changes between T2 and T3 in any group. However, a significant increase in this biomarker was reported for both groups (GS and GP) and was found between T3 and T4 (in the spring-–summer season). A significant decrease (*p* < 0.05) in RBC, PLTPLT, and TG was noted in GS, while RBC, HGBHGB, and HCT significantly decreased in GP. Moreover, a significant increase ofincrease in RBC was noted between T3 and T4 in GP. No other significant differences were reported for any of the groups. Significant time interactions were calculated for all the variables except WBC, TCTC, and LDL-C. No significant group x time× time interactions were found for any analyzed variable (Table 3). Finally, the significant (*p* = 0.023) but poor (*r *= −0.23) correlation between 25(OH)D and WBC was calculated (Figure 3). The higher level of 25(OH)D was related to a lower number of WBC.

**Table 3.** Changes in selected blood parameters in young soccer players in the supplemented (GS) and placebo group (GP) in the T2, T3T3, and T4.

Group	GS			GP			Inter-actions	*p*	pη2	OP
Time- point	T2	T3	T4	T2	T3	T4				
25(OH)D [ng/ml]	24.1 ± 5.23	26.6 ± 6.35	41.7 ± 6.95*†	24.8 ± 4.77	26.2 ± 4.52	39.5 ± 6.91*†	Time	<0.001	0.82	1.00
WBC (10^3^ [3[3]/µl)	6.63 ± 0.93	6.23 ± 1.23	6.17 ± 0.90	5.77 ± 1.05	5.37 ± 0.95	5.86 ± 1.28				
RBC (10^4^ [4]/µl)	5.34 ± 0.26	5.11 ± 0.27*	5.34 ± 0.34†	5.24 ± 0.42	5.03 ± 0.33*	5.18 ± 0.38	Time	<0.001	0.38	0.99
HGB (g/dL)	15.5 ± 0.71	15.1 ± 0.64	15.5 ± 0.73	15.4 ± 0.77	14.8 ± 0.59*	15.2 ± 0.72	Time	<0.001	0.37	0.99
HCT (%)	46.0 ± 1.89	44.9±1.92	45.9 ± 2.11	45.7 ± 2.10	43.8 ± 1.70*	45.1 ± 2.36	time	<0.001	0.28	0.96
PLT (10^3^ [3]/µl)	238.2 ± 33.87	216.3 ± 36.41*	223.5 ± 38.40	222.2 ± 33.79	210.9 ± 33.47	219.0 ± 47.79	Time	0.001	0.27	0.94
TC (mg/dL)	151.5 ± 27.94	148.9 ± 22.78	152.1 ± 28.81	141.2 ± 15.74	146.5 ± 22.25	141.6 ± 17.08				
HDL-C (mg/dL)	54.8 ± 10.54	58.5 ± 8.64	53.5 ± 9.73	57.2 ± 6.50	55.3 ± 7.36	52.5 ± 7.50	Time	0.011	0.19	0.79
LDL-C (mg/dL)	74.7 ± 18.10	76.8 ± 19.96	79.7 ± 20.63	64.7 ± 12.65	77.5 ± 22.06	74.2 ± 15.29				
TG (mg/dL)	110.1±57.74	65.7 ± 19.13*	96.5 ± 26.64	83.3 ± 37.21	63.0 ± 18.75	74.5 ± 31.08	Time	0.002	0.25	0.93
ALT (U/L)	22.4 ± 11.57	22.5 ± 8.61	18.0 ± 4.69	16.6 ± 4.63	20.5 ± 4.50	17.1 ± 5.38	Time	0.027	0.15	0.68
AST (U/L)	24.1 ± 5.17	35.8 ± 15.87	25.5 ± 6.04	21.7 ± 6.55	31.5 ± 7.64	25.8 ± 7.22	Time	<0.001	0.41	0.99
CK (U/L)	176.3 ± 70.21	598.8 ± 432.29	224.8 ± 108.50	184.6 ± 109.71	535.1 ± 241.28	311.2 ± 182.26	Time	<0.001	0.50	1.00

*Significantly different from T2; †Significantly different from T3; WBC-: white blood cells; RBC-: red blood cells; HGB-: hemoglobin; HCT-: hematocrit; PLT-: thrombocytes; TC-: total cholesterol; HDL-: C-high-density cholesterol; LDL-C-: low-density cholesterol; TG-: triglycerides; ALT-: alanine aminotransferase; AST-: asparagine aminotransferase; CK-: creatine kinase; pη2-: partial eta square; OP-: observed power.

## 4. Discussion

The main purpose of this research was to investigate the potential effect of vitamin D supplementation, sunlight radiationradiation, and home isolation on seasonal changes of 25(OH)D concentration and selected biomarkers during a one1-year training cycle in young soccer players. It was assumed that the highest drops in 25(OH)D concentration would occur in the autumn-–winter season when the sunlight radiation is low. Therefore, vitamin D supplementation could effectively prevent its deficiency and negative changes in related biomarkers. Furthermore, due to the unpredicted occurrence of the COVID-19 pandemic during the project, forced home isolation was recognized as an additional factor potentially influencing the results of analyzed blood parameters.

To our bestthe best of our knowledge, this is the first research analyzing this topic in a one1-year training cycle in young soccer players. Previous studies by Andelković et al. [36] and Saidi et al. [37] found variations of selected blood parameters in professional soccer players during three months3 months and six weeks6 weeks, respectively. Furthermore, My et al. [38] presented the most recent analyses involving selected biomarkers and physicalthe physical fitness of Italian Serie A soccer players during the COVID-19 pandemic.

The results presented in our research confirmed the hypothesis concerning the seasonal changes in 25(OH)D concentration. The highest drops of this biomarker were noted in the autumn-–winter seasons. However, the results obtained after home isolation did not confirm the hypothesis of the longitudinal and preventive effecteffects of vitamin D supplementation. The analysis of selected blood parameters exhibited significant variations during the project, and the differences between GS and GP were not statistically significant.

The seasonal changes of 25(OH)D concentration in athletes were previously demonstrated not only in the countries of Northern Europe [2,11,16,39] when insolation is relatively low but also in Southern Europe countries as well [7]. Wilson-Barnes et al. [12], who tested 47 university student-athletes from England (51.2°N), found that in October, the deficiency (≤20 ng/dl) of 25(OH)D concentration occurred in 43% of participants, while at the end of winter in 79%. Furthermore, García and Guisado [40] demonstrated that 57% of Spanish (41° N, 2°E) basketball players had a deficiency of 25(OH)D at the beginning of the spring. The results presented in the current study showed that the natural synthesis of vitamin D in athletes is highly affected by the season and related sunlight radiation. In T1 (September 2019), 83.3% of participants had optimal and 16.7% suboptimal levels of 25(OH)D concentration. Significant changes in this parameter were noted after three3 months (T2, December 2019), when 12.5% of the players had optimal and 62.5% had a suboptimal level of 25(OH)D. The deficiency of this biomarker was found in 25% of the participants. Despite the 8-week vitamin D supplementation (from half of January to half of March), no significant differences were found in T3 (half of May – measurements were delayed due to the pandemic occurrence) in the whole group (*n* = 24), nor in the GS and GP. The most favorable concentration of 25(OH)D was observed at the end of the summer season (T4) when only two participants had suboptimal and twenty-two optimal levels of this parameter. Based on the results from previous studies [9,15,21,41,42], it was assumed that an 8-week supplementation (5,000 IU/day) applied in GS during the winter season could have a significant effect on the level of 25(OH)D concentration in the tested young soccer players and effectively prevent its deficiency. However, the 2-month long home isolation (reduced sunlight exposure) that occurred after the supplementation period probably contributed to the lower level of 25(OH)D concentration. The results obtained in T3 were very similar in GS and GP (26.6 ± 6.35 ng/ml and 26.2 ± 4.52 ng/ml, respectively). The highest values of this biomarker were noted at the end of the project in T4 (an increase of 34.8% compared to T3). This improvement was probably caused by greater exposure to sunlight after the cancellation of the pandemic restrictions (from half of May to half of August). These results suggest that the effect of vitamin D supplementation does not last long, and constant dosing is needed during periods of deficiency (Table 3). This thesis was supported by Valtueña et al. [43], who reported that athletes who train indoors (e.g. handball, basketball, or volleyball players) and are not exposed to sunlight during training sessions obtain significantly lower values of 25(OH)D concentration than soccer players (who performed their sessions outdoor). Besides the sun radiation related to the season of the year and latitude [2,44], the daily diet is another important factor determining the acquisition of vitamin D. Kuchuk et al. [45], and Napiórkowska et al. [46] stated that recommended daily intake of vitamin D for people leaving in Poland should be 800–2000 IU/day. Moreover, Rusińska et al. [1] and Jurek et al. [47] demonstrated that the daily intake of products rich in vitamin D is insufficient in the Polish population and includes no more than 200 IU/day. The results presented by Brustad et al. [14] confirmed the high effectiveness of the diet in covering the demand for vitamin D during periods of its deficiency. Those participants of this research who applied a diet rich in oil products and fatty fish (salmon, mackerel, and herring) reached even higher 25(OH)D concentration in winter than in summer. Our previous research [31] showed that young soccer players’ average daily vitamin D intake was between 145 and 187 IU/day. Taking into account the suggestions of Cashman et al. [48], who claimed to consume at least 400–1040 IU/day, it can be stated that the diet of our players significantly varied from these recommendations. Our results were similar to those of Bacx et al. [49], who tested 128 athletes from the Netherlands Olympic Team. The daily vitamin D intake in this group was 168 ± 104 IU/day. Even lower values (139 ± 78 IU/day) were reported in professional basketball players [40]. Therefore, vitamin D supplementation seems to be the primary source of preventing its deficiency, especially in athletes who train indoors or were forced into home isolation during the COVID-19 pandemic [50]. Moreover, according to Grant et al. [51], supplementation could reduce the risk of Sars-CoV2 virus infection. From this point of view, our supplementation activities were preventive, although due to the study purpose, and it had not of a clinical nature.

Several biomarkers related to seasonal changes in 25(OH)D, morphology, lipid profileprofile, and muscle damage were investigated in this research. In the previous research from this project [31], the bone resorption markers were also discussed. Considering the relations between the measured biomarkers, we found a significant negative (*r* = −0.23) correlation only between 25(OH)D concentration and WBC, suggesting that players with lower vitamin D levels have a higher number of white blood cells. This relationship is in line with the results presented by Calton et al. [52], who found that the increase of 25(OH)D concentration during the summer season was accompanied by a significant decrease in inflammatory indicators such as CRP, TNF-α, IL-6IL-6, or IL-12. Similarly, Hashemi et al. [53] confirmed that the participants of their experiment, with the increase in 25(OH)D, were characterized by a more significant activity of anti-inflammatory interleukins (IL-27, TGF-β1,TGF-β1, and IL-10) and a decrease in the activity of pro-inflammatory interleukins IL-17A and IL-6. In the current study, the lowest number of WBC was observed in T2 (end of autumn, increase of 5.19% in comparison to T1), and the lowest values were found in T4 (end of summer, decrease of 4.00% in comparison to T3). Thus, these changes seemed to be affected by the applied high training loads (competitive season) than by changes in 25(OH)D concentration. However, during the summer season, these changes were less dynamic (Table 2). After dividing the participants into subgroups, we calculated in T4 a larger drop of WBC in GS (6.94%) than in GP (1.14%, Table 3). We suppose that, although the effect of supplementation was not significant, it could positively influence reducing the pro-inflammatory effect in intensively training athletes.

More dynamic changes in four time points along the one1-year training cycle were registered in RBC and HGB. The significant increase of HGB at the end of autumn (T2, *n* = 24) at a low concentration of 25(OH)D confirms the thesis ofby Skalska et al. [15] and Andzelković et al. [36] that training loads could affect these parameters more than seasonal changes of 25(OH)D. Furthermore, My et al. [38], who tested soccer players during the COVID-19 pandemic, confirmed our observations (results obtained in T3 and T4) that applied at this time training loads were insufficient to improve biomarkers of cellular oxygenation. Considering the changes in time of the analyzed blood oxygenation indicators, we found a significant (*p* < 0.001) time interaction and fair effect size for GS and GP (*p*η [1] = 0.38 and 0.37, respectively). However, no group or group x time× time effect was exhibited. Therefore, it can be assumed that the 8-week vitamin D supplementation had no significant effect on improving RGB and HGB in the tested athletes (Table 2). According to Rampinini et al. [27], the individual home-based training performed by the players during the 2-month isolation could have been an additional stimulus supporting the process of cellular oxygenation.

Systematic monitoring of blood parameters allows applying the training loads precisely and avoids the detraining effect in professional soccer players [54]. Therefore, we decided to control the participants’ lipid profiles and muscle damage parameters in our project. The mechanism regulating the lipid profile in people with different levels of 25(OH)D and supplemented with vitamin D is ambiguous in athletes and untrained people. The cohort study ofby Ponda et al. [55] demonstrated that patients with vitamin D deficiency (<20 ng/ml) had lower average values of TC, LDL-C, and TG and higher values of HDL-C in comparison with optimal leveloptimal-level patients (≥30 ng/ml). The increase ofin vitamin D (through the supplementation) was related to an increase in TC and HDL-C, while the changes in LDL-C and TG were not significant. The authors stated that correcting vitamin D deficiency did not affect clinical changes in lipids. Li et al. [56], taking into account the vitamin D supplementation, observed that large cohorts of participants with an annual increase of 25(OH)D concentration showed a tendency to decrease in TC, LDL-CC, and TG. On the other hand, in participants with an annual decrease of 25(OH)D concentration, the increment of these parameters was noted. Furthermore, the authors proved that 25(OH)D variations were related to changes in HDL-C. Jastrzębska et al. [23] demonstrated that an increase of 25(OH)D concentration had no significant effect on the lipid profile of young soccer players during an 8-week supplementation.

The results of the current study did not exhibit any significant changes in the lipid profile of the tested players (*n*-24) during the analyzed one1-year training period (Table 2). However, after dividing the participants into subgroups, significant time interaction was found in HDL-C (*p* < 0.001) and TG (*p* < 0.002). Moreover, TG significantly decreased in GS between T2 and T3, suggesting the positive influence of vitamin D supplementation or low-intensity training loads applied during the preseason period. However, in our opinion and based on OlivieraOliveira et al. [57], the increase ofincrease in TG in T4 can be explained by a potentially larger intake of high-carbohydrate drinks during the competitive period, when players perform more high-intensity training loads (Table 3).

Creatine kinase is one of athletes’ most widely used muscle damage markers [58,59]. Considering the values of CK in the whole group (*n* = 24), we found a significant decrease in the activity of this biomarker at the end of the competition season (T1-T2, −160.9 U/L) and after the preseason period (T3÷T4T3 ÷ T4, −292.7 U/L). Although thea significant (*p* < 0.001) time interaction was calculated, no significant differences in CK were noted after dividing the players into GS and GP. Therefore, the more dynamic drop in CK values between T3 and T4 in GS (−374.0 U/L) than in GP (−223.9 U/L) may be a symptom of a possible effect of supplementation in combination with an endogenous synthesis of vitamin D during springduring the spring and summer. This thesis is supported by Żebrowska et al. [60], who observed that a higher level of 25(OH)D concentration after a 43-week supplementation of vitamin D (2000 IU/day) increases the preventive effect of eccentric exercises in long-distance runners. However, the significant time interaction may suggest that lowerthe lower CK concentration in the tested players is rather the result of muscle adaptation to the applied training loads than 25(OH)D variations. This statement is partially confirmed by Meyer and Meister [58] who showed in their meta-analysis that high-intensity interval training causes the increase ofan increase in CK concentration in the blood plasma of soccer players.

Furthermore, Radzimiński et al. [59] exhibited that even a 2-week sports camp enhances the levels of SC and AST in professional soccer players. Both these publications proved that high-intensity training (including eccentric exercises) improves muscle adaptation to eccentric exercise. Therefore, such training reduces muscle damage, which results in a lower level of CK 24–48 hours48 h after implementing such effort.

Previous research [61] suggested that three months3 months of vitamin D supplementation (3200 IU/day) could decrease the activity of AST. However, analyzing the changes ofin liver enzymes, such as ALT Iand AST, we found that only changes in AST were similar to CK. In our opinion, also, in this case, these changes are instead affected by soccer training adaptation than by vitamin D supplementation.

Biomarkers such as PTH, PP, and Ca are important parameters that complemented the results of our annual project and were described in the previous study [31]. It was reported that a high 25(OH)D concentration was related to low PTH. Furthermore, a large drop of 25(OH)D (between T1 and T2) corresponded to a significant increase in Ca and P. Between December and May, when despite the supplementation in GS, the level of 25 (OH)D was low, we registered a significant increase in PTH with a simultaneous decrease in Ca and P. It is possible that the effect of home isolation and reduced effect of supplementation. At the final stage of the project (T4), a significant increase of 25(OH)D was accompanied by a significant decrease in PTH, which can be considered a positive metabolic effect.

In the present research project, some advantages and one limitation are worth mentioning. In our opinion, the study duration and homogeneity of the group (players from one club, who were analyzed in terms of their nutrition) with the use of a double-blind design could be considered strengths of the current investigation. The main limitation of the project was the occurrence of the COVID-19 pandemic, which made it impossible to perform all the measurements according to the original design. The number of participants who completed the project was relatively low (*n* = 24), especially after dividing them into GS and GP (*n* = 11, and *n* = 13, respectively). However, this fact could be justified because most soccer teams involve no more than 25–30 players. Therefore, recruiting a larger number of athletes was impossible due to design restrictions (identical training loads and localization).

## 5. Conclusions

The results presented in this research confirmed the hypothesis of seasonal changes in 25(OH)D concentration during four seasons. It was exhibited that these changes are dependent on the sunlight radiation expressed in UV units. The applied 8-week vitamin D supplementation had no extended effect on the level of 25(OH)D concentration. Moreover, the home isolation due to the COVID-19 pandemic reduced the athletes’ sunlight exposure and could negatively influence endogenous vitamin D synthesis. Although the seasonal changes in 25(OH)D concentration were significant, they did not affect most of the analyzed biomarkers. Only a number of WBC was significantly related to 25(OH)D concentration. Changes in parameters such as HGB, ASTAST, and CK were statistically significant for the whole group (*n* = 24). In our opinion, the recorded significant changes in some of the biomarkers in GS and GP were rather the result of the applied training loads than vitamin D supplementation or seasonal changes of 25(OH)D concentration.

## Data Availability

The data presented in this study are available on request from the corresponding author.
